# Changes in Electroencephalography Complexity using a Brain Computer Interface-Motor Observation Training in Chronic Stroke Patients: A Fuzzy Approximate Entropy Analysis

**DOI:** 10.3389/fnhum.2017.00444

**Published:** 2017-09-05

**Authors:** Rui Sun, Wan-wa Wong, Jing Wang, Raymond Kai-yu Tong

**Affiliations:** ^1^Division of Biomedical Engineering, Department of Electronic Engineering, Chinese University of Hong Kong Hong Kong, Hong Kong; ^2^School of Mechanical Engineering, Xi'an Jiaotong University Xi'an, China

**Keywords:** electroencephalography, motor observation, stroke, complexity, fuzzy approximate entropy

## Abstract

Entropy-based algorithms have been suggested as robust estimators of electroencephalography (EEG) predictability or regularity. This study aimed to examine possible disturbances in EEG complexity as a means to elucidate the pathophysiological mechanisms in chronic stroke, before and after a brain computer interface (BCI)-motor observation intervention. Eleven chronic stroke subjects and nine unimpaired subjects were recruited to examine the differences in their EEG complexity. The BCI-motor observation intervention was designed to promote functional recovery of the hand in stroke subjects. Fuzzy approximate entropy (fApEn), a novel entropy-based algorithm designed to evaluate complexity in physiological systems, was applied to assess the EEG signals acquired from unimpaired subjects and stroke subjects, both before and after training. The results showed that stroke subjects had significantly lower EEG fApEn than unimpaired subjects (*p* < 0.05) in the motor cortex area of the brain (C3, C4, FC3, FC4, CP3, and CP4) in both hemispheres before training. After training, motor function of the paretic upper limb, assessed by the Fugl-Meyer Assessment-Upper Limb (FMA-UL), Action Research Arm Test (ARAT), and Wolf Motor Function Test (WMFT) improved significantly (*p* < 0.05). Furthermore, the EEG fApEn in stroke subjects increased considerably in the central area of the contralesional hemisphere after training (*p* < 0.05). A significant correlation was noted between clinical scales (FMA-UL, ARAT, and WMFT) and EEG fApEn in C3/C4 in the contralesional hemisphere (*p* < 0.05). This finding suggests that the increase in EEG fApEn could be an estimator of the variance in upper limb motor function improvement. In summary, fApEn can be used to identify abnormal EEG complexity in chronic stroke, when used with BCI-motor observation training. Moreover, these findings based on the fApEn of EEG signals also expand the existing interpretation of training-induced functional improvement in stroke subjects. The entropy-based analysis might serve as a novel approach to understanding the abnormal cortical dynamics of stroke and the neurological changes induced by rehabilitation training.

## Introduction

Stroke is the leading cause of adult disability worldwide. In the United States, more than 700,000 people experience a stroke each year. Among them, 25% will die and 15–30% will remain with a physical disability (Lethbridge-Çejku and Vickerie, 2005). Many of these patients show moderately favorable recovery at the shoulder and elbow, but limited motor restoration at the wrist and hand joints. After 6 months, around 38% show some recovery in hand function, but only 11.6% achieve complete functional recovery of dexterity (Kwakkel et al., 2003). Recovery of hand function after stroke is crucial to perform activities of daily life but is always the most challenging aspect to be achieved.

Intensive therapeutic interventions could contribute to significant improvement in the functional use of the affected parts after chronic stroke (Lum et al., 2002). A brain–computer interface (BCI) is one approach by which the movement intentions of a patient can be interpreted (Pfurtscheller and Neuper, 2001), whereas motor imagery (MI) is the mental rehearsal of a kinematic movement (Belda-Lois et al., 2011). Many studies have combined these two techniques and applied them to post-stroke rehabilitation (Pfurtscheller and Neuper, 1997; Ang et al., 2010; Teo and Chew, 2014). However, some recent reports have shown that the effect of BCI-MI on motor recovery is debatable. There are mainly two reasons: First, it is difficult to perform MI without adequate practice; thus, the effects of training vary greatly from person to person owing to variations in MI interpretation. One way to overcome this limitation is to adopt motor observation. Aziz-Zadeh et al. (2006) reported that the mirror neuron system (MNS), a parieto-frontal neural network distributed in the human brain, can be activated both when individuals perform a particular action and when they observe a similar action being performed by others. Therefore, observation of the movement of another person could activate the same motor areas as those activated during MI practice, and thus reduce the variance in MI interpretation among different individuals. Second, the lack of real-time. In 2004, Muthukumaraswamy et al. (Muthukumaraswamy and Johnson, 2004; Muthukumaraswamy et al., 2004) reported on electroencephalogram (EEG) mu (8–13 Hz) suppression during observation of a motor action performed by another person. Mu suppression was defined as the ratio of the power during the stimulus condition relative to the power during the resting baseline condition, with the assumption that mu synchrony would be greatest during the non-biological observation, owing to the lack of movement or perception of movement, but should be suppressed while observing biological action (Oberman et al., 2008). Mu suppression was represented as an indirect measure of the recruitment of MNS activity (Muthukumaraswamy et al., 2004) and could be used as a dependent variable to give feedback to the subject. In this study, BCI, motor observation, and MNS theory were integrated to design a novel rehabilitation system that is expected to overcome the potential problems in existing BCI and MI systems and facilitate the restoration of hand function in stroke patients.

Clinical assessment scales are routinely used to assess residual motor function after stroke. However, kinematic and kinetic indexes only reflect external motor performance, whereas the internal mechanism of neurological changes could determine external motor performance. The EEG is a recorded signal of the overlapped oscillations of brain cell action potentials in time and space. Many linear EEG-derived indexes, such as frequency analysis and EEG topography (Nuwer et al., 1987), have been used as evaluation measures. However, the brain is a complex nonlinear system, and the EEG signal is demonstrated nonlinearly at the neuronal level. Therefore, it would be more appropriate to use nonlinear methods to analyze EEG signals (Klonowski, 2009). Moreover, a nonlinear dynamic approach could provide novel insights into brain diseases and could be a useful tool in understanding the mechanisms of neuronal plasticity after injury and during rehabilitation. C. J. Stam summarized many kinds of nonlinear time series analysis. For example, the dimension is used to estimate the degrees of freedom of the system, Lyapunov exponents and entropy is used to reflect unpredictability of the dynamics due to the sensitive dependence on initial conditions (Stam, 2005). Studies that are concerned with effects of stroke from this perspective are less numerous but there are some; for example, the point correlation dimension was used for the analysis of the EEG recorded in patients with unilateral stroke caused by middle cerebral artery occlusion (Molnar et al., 1999). However, a relatively large dataset is needed for these nonlinear dynamic measures to obtain reliable and consistent results, or spurious results could be generated. Approximate entropy (ApEn) is a powerful nonlinear method for the characterization of short physiological signals (less than 1,000 points) (Pincus, 1991; Yentes et al., 2013). The ApEn method was first developed by Pincus (Pincus, 1991), who aimed to measure the regularity of a time series. It has been used to analyze the EEG to assess the conscious state (Wu et al., 2011), characterize Alzheimer's disease (Cao et al., 2015), and elucidate the pathophysiological mechanisms in schizophrenia (Takahashi et al., 2010). Recently, fuzzy approximate entropy (fApEn) has been developed which is a combination of the concept of “fuzzy sets” introduced by Zadeh (1982) and ApEn introduced by Pincus (1991). Xie et al. (2011) further confirmed the advantage of fApEn compared to ApEn during a complexity analysis of electromyography (EMG). Ao et al. (2015) also demonstrated the consistency and robustness to noise of fApEn in the analysis of EMG signals, in comparison to ApEn and sample entropy. Considering its successful application in the analysis of short noisy physiological signals, fApEn was used in this study to investigate the changes in complexity of EEG signals in post-stroke patients after rehabilitation training. We hypothesized that the complexity of EEG signals would change in association with training-induced upper limb motor function improvement in stroke patients.

## Materials and methods

### Subjects

Eleven post-stroke subjects (nine males and two females; aged 55.89 ± 9.24 years) and nine unimpaired subjects (seven males and two females; aged 30.45 ± 6.60 years) were recruited. The data of age and results of clinical assessments are presented as mean ± standard error (SE). The time since stroke (TAS) of all subjects exceeded 1 year. So, the natural recovery is too slow to cause a significant improvement in motor function during the period of training (2–3 months). Table 1 summarizes the demographics of the stroke subjects and Table 2 summarizes the lesion location of these subjects. Three subjects did not do fMRI scan due to the health problem. The inclusion criteria comprised of the following: (1) sufficient cognition to follow simple instructions and understand the purpose of the experiment (Mini-Mental State Examination > 21); and (2) hemiparesis resulting from a unilateral brain lesion with time since stroke greater than 6 months before study enrollment. The exclusion criteria comprised the following: (1) severe hand spasticity; (2) open hand wound or hand deformity; and (3) visual field deficits. The recruited healthy subjects have sufficient cognition to follow simple instructions and understand the purpose of the experiment (MMSE > 21). All subjects had been required to not participate another rehabilitation project during this training period and given written informed consent according to the Declaration of Helsinki. The Joint Chinese University of Hong Kong-New Territories East Cluster Clinical Research Ethics Committee (CUHK-NTEC CREC) approved the experimental protocol (agreement #2014.705-T). This study was also registered at www.clinicaltrials.gov, with the study identifier NCT02323061. Clinical assessment scales used in the present study included the Upper Limb Fugl-Meyer Assessment (Fugl-Meyer et al., 1975) (UL-FMA) (range: 0–66); Action Research Arm Test (Carroll, 1965) (ARAT) (range: 0–57); and Wolf Motor Function Test (Wolf et al., 2001) (WMFT) (range: 0–75). The clinical scales of each stroke subject before and after the training are presented in Table 1.

**Table 1:** Basic information of 11 post-stroke subjects.

**Subject**	**Gender**	**Age**	**Type**	**Affected hand**	**TSS**	**UL-FMA**		**ARAT**		**WMFT**	
						**Pre**	**Post**	**Pre**	**Post**	**Pre**	**Post**
1	M	58	Isch	R	10	22	24	15	14	33	36
2	F	48	Isch	L	1	36	41	8	20	29	38
3	M	59	Isch	L	11	25	33	28	38	25	34
4	M	65	Hemo	R	8	24	29	10	21	34	36
5	M	46	Isch	L	1	20	34	3	21	26	39
6	M	66	Hemo	L	1	13	16	8	15	17	21
7	F	68	Isch	L	3	25	26	14	27	36	42
8	M	46	Hemo	L	1	17	25	16	17	23	28
9	M	47	Hemo	R	2	20	24	15	29	19	27
10	M	59	Isch	L	3	17	14	4	6	16	20
11	M	52	Isch	R	1	41	36	32	27	43	43
Mean		55.82			3.82	23.64	27.45	13.91	21.36	27.36	33.09
SD		8.41			32.89	8.30	8.23	9.14	8.66	8.55	9.97

*Hemo, hemorrhagic stroke; Isch, ischemic stroke; M, male; F, female; R, right; L, left; TSS, time since stroke; UL-FMA, Upper-Limb Fugl-Meyer Assessment (maximum: 66); ARAT, Action Research Arm Test (maximum: 57); WMFT, Wolf Motor Function Test (maximum: 75)*.

**Table 2:** Lesion locations of eight post-stroke subjects.

**Subject**	**Lesion location**
1	L PLIC, putamen
2	R putamen, insula
3	
4	L insula, putamen, IFG, temporal pole
5	R MFG, SFG, precentral, supramarginal, SMA
6	R insula, ITG, IOG, putamen
7	
8	R ITG, MTG, STG, MOG, angular, supramarginal
9	
10	R insula, putamen, rolandic operculum, IFG
11	L MFG, precentral, IFG, postcentral, insula, SFG

*L, left hemisphere; R, right hemisphere; M, male; F, female; PLIC, posterior limb of internal capsule; IFG, inferior frontal gyrus; MFG, middle frontal gyrus; SFG, superior frontal gyrus; SMA, supplementary motor area; ITG, inferior temporal gyrus; MTG, middle temporal gyrus; STG, superior temporal gyrus; IOG, inferior occipital gyrus; MOG, middle occipital gyrus*.

### BCI-motor observation training system

**Figure 1:**
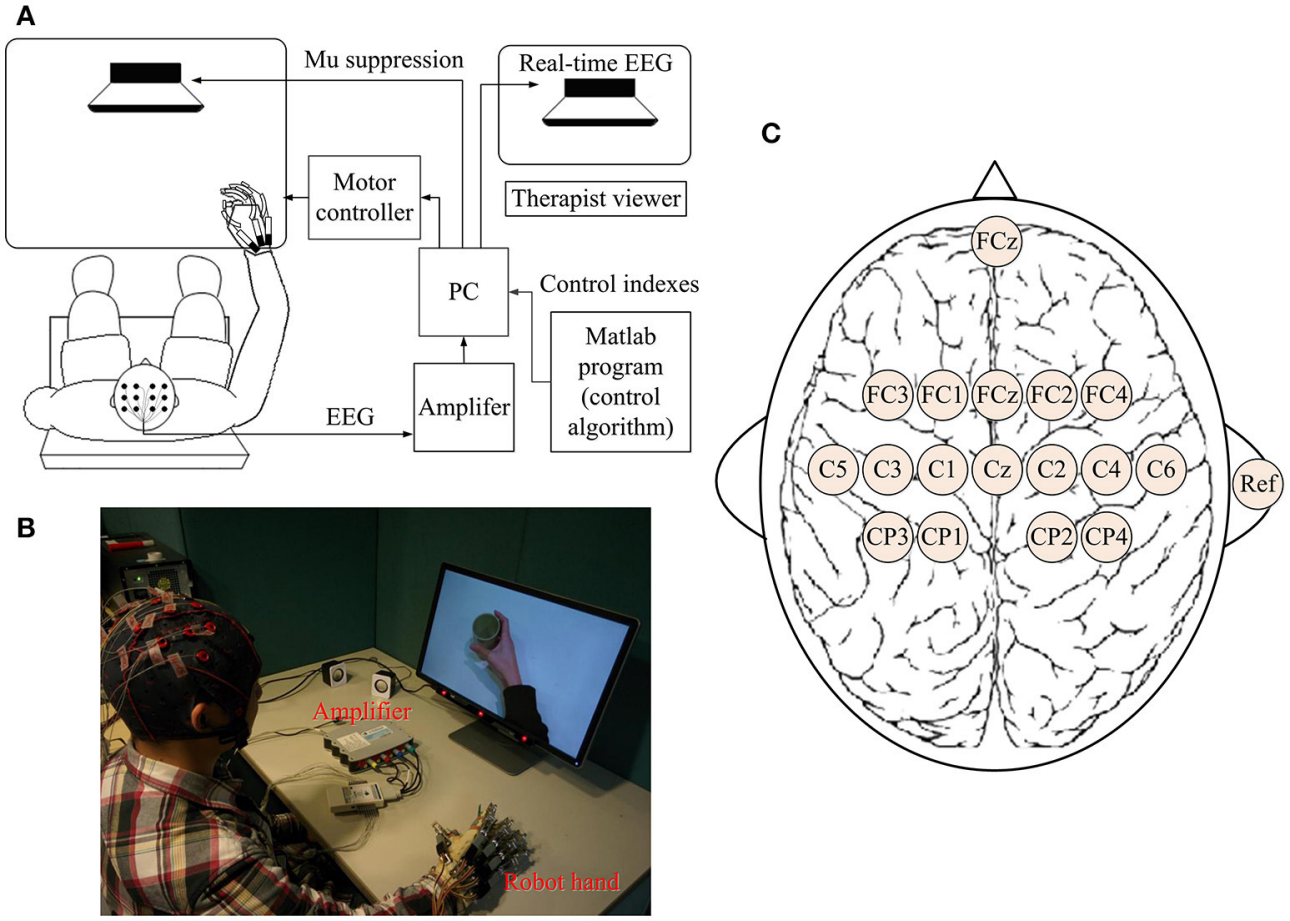
Schematic diagram **(A)** and real situation **(B)** of the BCI-motor observation training system for hand training. **(C)** The location of electrodes.

A BCI-motor observation training system was developed as shown in Figure 1A. The EEG signals of each subject were captured by 16 active electrodes (g.LADYbird, g.Tec Medical Engineering GmbH, Austria) and amplified by an amplifier (g.USBamp, g.Tec Medical Engineering GmbH, Austria), and then processed by a computer. A paradigm was played in a fixed sequence to guide the subject to complete a training task while a video-based stimulus was provided. A robotic hand (Tong et al., 2013) was used to assist the paretic hand to grasp/open, based on the result of the mu suppression algorithm that was calculated from the EEG signals captured during the video. The system also displayed a mu suppression score on the computer screen that was placed in front of the subject to provide real-time feedback. The mu suppression score provides information about the degree of activation of the MNS, and the subject could adjust the motion observation according to this index in order to achieve higher scores. It will be described in detail in Section Mu Suppression Score. After all tasks were completed, the computer recorded the EEG signals and time-marks for further analysis.

### EEG acquisition

EEG signals were referenced to a unilateral earlobe, grounded at a frontal position (Fpz), and sampled at 256 Hz using 16 active electrodes with the location as shown in Figure 1C. EEG signals were also processed in real-time using a band-pass filter (2–60 Hz) and a notch filter (48–52 Hz) to remove artifacts and power line interference, respectively. All electrodes were filled properly with a conductive gel to ensure the transmission impedance remained below 1 kOhm. The EEG electrodes were placed over the central area according to the international 10–20 system. Consistent with the methods of similar studies (Pineda, 2005; Oberman et al., 2013), EEG signals from C3 and C4 electrodes were used for BCI control. Furthermore, some of the sites that are related to motor function were used for offline analyses (left hemisphere: FC3, C3, and CP3; right hemisphere: FC4, C4, and CP4). The FC3/FC4 lies over the pre-motor cortex and the C3/C4 lies over the primary motor cortex. CP3/CP4 corresponds to the supramarginal gyrus that is part of the somatosensory association cortex. These six electrodes covered the major part of the mirror neuron system (Carlson, 1994).

### Experiment and paradigms

All stroke subjects received BCI-motor observation training consisting of 20 sessions, with an intensity of 3–5 sessions per week that was completed within 5–7 weeks. Healthy subjects were only required to participate one session. During each session, each subject sat in a height-adjustable chair with his/her: (1) shoulder positioned at 90° abduction; (2) elbow flexed at 90°; (3) arm pronated, such that the palm was directed medially; and (4) wrist positioned neutrally without any flexion/extension, as shown in Figure 1B. The non-dominant arm was used for the healthy subject while the affected arm was used for stroke subject. A cushion was used to support and maintain the position of the subject's arm.

**Figure 2:**
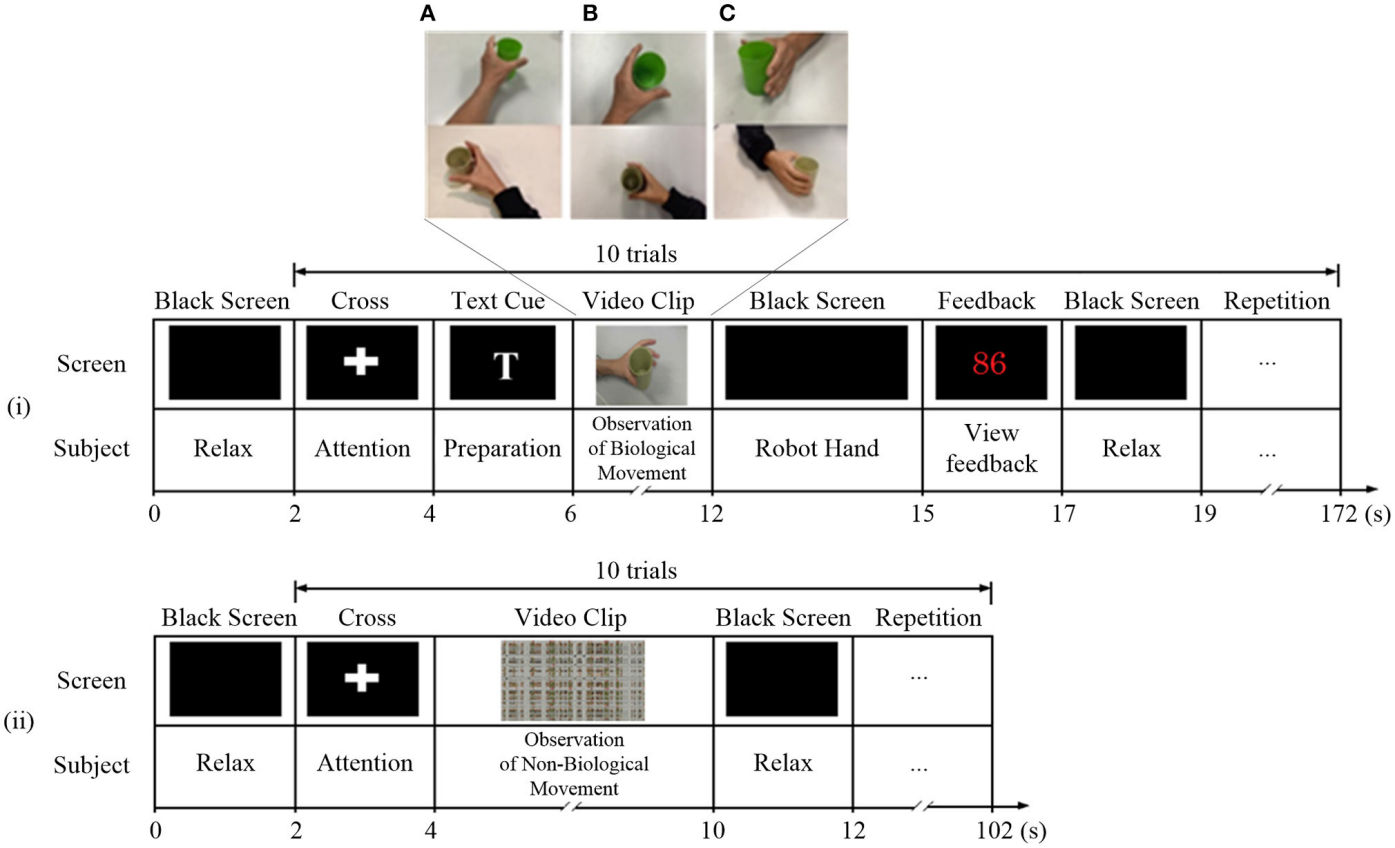
Experiment paradigms for observation of **(i)** biological and **(ii)** non-biological movements. During the observation of biological movements, video clips were displayed in **(A)** egocentric, **(B)** overhead, and **(C)** allocentric perspectives.

Two paradigms were used (Figure 2): (1) observation of biological movement: observation of a video demonstrating grasping or releasing of a cup using the affected hand, from three different perspectives (A: egocentric, B: overhead, and C: allocentric). In the egocentric viewpoint, the subject viewed the actions from the first-person perspective (Figure 2A). In the overhead viewpoint, the subject viewed the actions from above (Figure 2B). In the allocentric viewpoint, the subject viewed the actions from the third-person perspective (Figure 2C). (2) Observation of non-biological movement: observation of a video generated by decomposition of the video clip of biological movement into frames (24 frames per second), with each frame spatially scrambled (192 × 108 fragments in each frame), to ensure that the hand action could no longer be recognized. The timings of the experimental sequence for observation of biological and non-biological movements are shown in Figure 2. During the observation of biological movements (Figure 2i), a dark screen was first displayed for 2 s, followed by a white cross for 2 s. A text cue of “hand grasp” or “hand open” was then displayed for 2 s. A video clip with a duration of 6 s was then displayed. Subjects were asked to observe the actions and avoid blinking. The mu suppression score was calculated based on the EEG signals during the video clip (introduce in Section Mu Suppression Score). A robotic hand provided mechanical support to assist the subject in the completion of the hand grasp/open task during the following 3 s, if the mu suppression score was above 20. Such scores meant that the ratio of the mu power between biological and non-biological observation was below 80% according to the average results reported in Perry's study (Perry and Bentin, 2009). The mu suppression score was then shown for 2 s. The trial ended with the display of a dark screen for 2 s. During each session, the trial was repeated 100 times and video clips of the grasping hand and opening hand were shown alternately with random viewing perspectives. Subjects were allowed to rest for 1 min after 10 trials. The sequence of the experimental paradigm for observation of non-biological movements (Figure 2ii) included only a black screen, cross, scrambled video clips, and then another black screen. The display of experimental sequences for the two paradigms was controlled by the Psychophysics Toolbox 3.0 (http://psychtoolbox.org/) (Brainard, 1997; Pelli, 1997).

### Mu suppression score

Mu suppression reflects an event-related desynchronization (ERD) of the EEG caused by an increase in neural activity (Kuhlman, 1978). Many researchers believed that mu suppression is associated with the activation of MNS in human brains (Bartur et al., 2015). To compute mu suppression, C3 or C4 was selected according to the subject's ipsilesional side. The EEG data were converted to the frequency domain by a fast Fourier transform algorithm with a Hanning window covering the EEG data during the video period (6 s) in paradigm. The mean power in the mu band (8–13 Hz) for the selected electrode was calculated. The value of mu suppression score is equal to the negative difference of mu power between the observation of biological movement and non-biological movement, divided by the mu power during observation of non-biological movement (baseline), and multiply by 100 (Oberman et al., 2008; Braadbaart et al., 2013), as expressed by the following equation:

$$MuSC=-\frac{MuP_{bo}-MuP_{nbo}}{MuP_{nbo}}*100$$

where *MuSC* represents the Mu suppression score, *MuP*_*bo*_ represents the mu power of EEG during observation of biological movement, and *MuP*_*nbo*_ represents the mu power of EEG during observation of non-biological movement.

### Fuzzy approximate entropy (fApEn)

EEG complexity has been used to assess Alzheimer's disease (Cao et al., 2015), schizophrenia (Takahashi et al., 2010) and epilepsy (Acharya et al., 2012), it may reflect the condition of neuronal death, loss of synaptic connections, and the general effects of neurotransmitter deficiency. In addition, compared to the traditional linear approaches, EEG complexity as a nonlinear dynamic approach provides novel insights into brain diseases. This study adopted the fApEn algorithm described in a previous study (Ao et al., 2015). A brief introduction to the algorithm is described in this section. To compute the fApEn of an N sample series {*u*(*i*) : 1 ≤ *i* ≤ *N*}, a vector of length m could be derived from the time series:

(1) $$X_{i}^{m}=\left\{u(i),\cdots ,u(i+m-1)\right\}-\frac{1}{m}\sum_{j=0}^{m-1}u(i+j)$$

Where $\frac{1}{m}\sum_{j=0}^{m-1}u(i+j)$ is the baseline of the vector.

The distance $d_{ij}^{m}$ between $X_{i}^{m}$ and $X_{j}^{m}$ was defined as:

(2) $$d_{ij}^{m}=\underset{k\in (0,m-1)}{\max}\left|w(i+k)-w_{0}(i)-u(j+k)+u_{0}(j)\right|$$

A fuzzy function $D_{ij}^{m}\left(n,r\right)$ was formulated to calculate the similarity degree of the two vectors $X_{i}^{m}$ and $X_{j}^{m}$. where *n* and *r* are two parameters that determine the width and gradient of the boundary of the exponential function, respectively:

(3) $$D_{ij}^{m}(n,r)=\exp(-{\left(d_{ij}^{m}/r\right)}^{n})$$

The function ϕ^*m*^ then aggregated the similarity from any vector in the time series to another as follows:

(4) $$\phi^{m}(n,r)=\frac{1}{N-m}\sum_{i=1}^{N-m}\left(\frac{1}{N-m-1}\sum_{j=1,j\neq i}^{N-m}D_{ij}^{m}\right)$$

Finally, fApEn (*m, n, r, N*) was estimated by the algorithm of the difference between the function of the length m + 1 and m.

(5) $$fApEn(m,n,r,N)=\ln\phi^{m}(n,r)-\ln\phi^{m+1}(n,r)$$

### Statistical analysis

Statistical analysis was performed using the IBM SPSS 22 software (SPSS Inc., Chicago, Illinois, USA). The Wilcoxon signed-rank test was applied to verify the statistical significance of changes in the UL-FMA, ARAT, and WMFT between pre- and post-training. For fApEn and mu power analyses, two-way repeated-measures analysis of variance (ANOVA), with time (pre-training vs. post-training) and electrode (FC3, C3, CP3, FC4, C4, and CP4) as within-subject factors were used to assess the training effects. Two-way mixed analysis of covariance (ANCOVA), with group (stroke: pre- or post-training vs. healthy control) as between-subject factor, electrode (FC3, C3, CP3, FC4, C4, and CP4) as within-subject factor, and age as covariate, was performed to test for group differences. The Greenhouse-Geisser adjustment was applied to the degrees of freedom for all analyses if the Mauchly's test of sphericity was significant. Spearman's correlation analysis was used to investigate potential associations between fApEn and clinical scales (UL-FMA, ARAT, and WMFT). The significance level for all statistical analyses was set at *p* < 0.05.

## Results

### Clinical scales

The results from the clinical scales are presented in Table 1. The Wilcoxon signed-rank test showed a significant increase in the group mean WMFT score (pre-WMFT: 27.36 ± 8.55; post-WMFT: 33.09 ± 9.97; *p* = 0.005); the group mean ARAT score (pre-ARAT: 13.91 ± 9.14; post-ARAT: 21.36 ± 8.66; *p* = 0.014); and the group mean UL-FMA score (pre-UL-FMA: 23.64 ± 8.30; post-UL-FMA: 27.45 ± 8.23; *p* = 0.045) after training.

### Mu suppression

**Figure 3:**
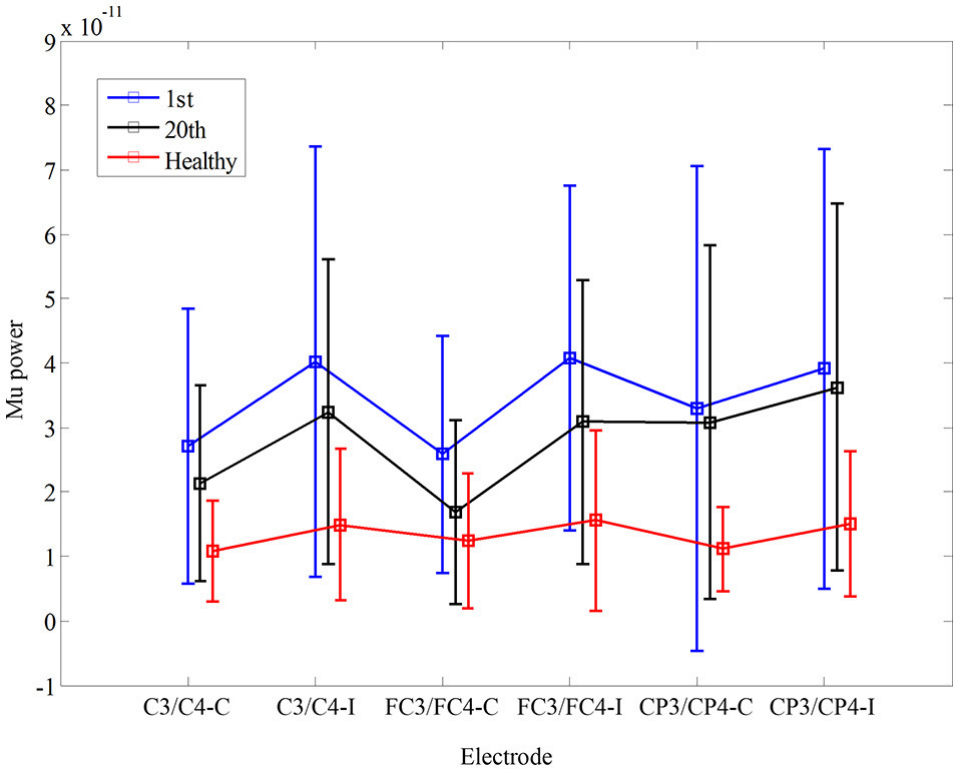
Comparison of mu power during non-biological observation at six electrodes (C3, C4 FC3, FC4, CP3, CP4) among healthy subjects and stroke subjects at pre- and post-training.

In Figure 3, there was no significant interaction between the time and electrode, *F*_(3.085, 30.850)_ = 1.447, *p* = 0.248. Furthermore, there was no significant main effect of the time [*F*_(1, 10)_ = 1.184, *p* = 0.302], and the electrode [*F*_(1.369, 13.693)_ = 1.342, *p* = 0.281]. There was no significant difference in mu power of the electrodes between healthy controls and stroke patients before the training, *F*_(1, 17)_ = 0.02, *p* = 0.968. Also, there was no significant difference in fApEn of the electrodes between healthy controls and stroke patients after the training, *F*_(1, 17)_ = 0.042, *p* = 0.840.

### EEG fApEn

**Figure 4:**
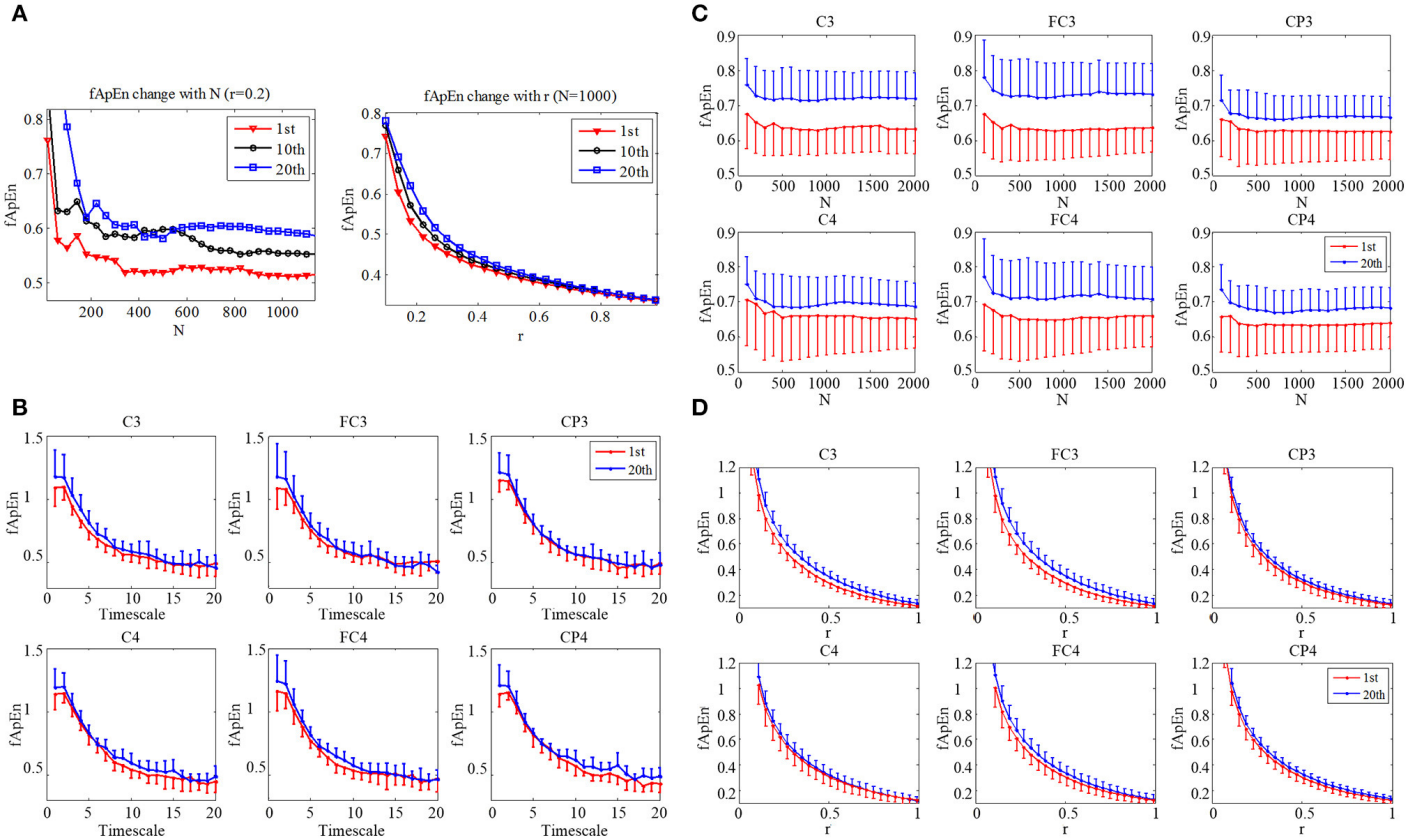
**(A)** Change of EEG fApEn with increase of window N, and tolerance r in the 1st, 10th, and 20th training session in a subject (S3). **(B)** Average multiscale fApEn of EEG of 11 stroke subjects at six electrodes (C3, C4, FC3, FC4, CP3, CP4). Average change of EEG fApEn with increase of **(C)** window N and **(D)** tolerance r of 11 stroke subjects at six electrodes.

The fApEn parameter choices of window length N and tolerance window r were tested with the EEG data from a stroke participant (S3) as in Figure 4A. The window N increase from 40 points to 1200 points in steps of 40 points. The EEG signal was picked from C3 during motor observation in three training sessions: the 1st, 10th, and 20th sessions. The fApEn curve from the 20th session was larger than that from the 10th session, and the fApEn from the 10th session was larger than that from the 1st session. There were only a few crossovers between the sessions when N was less than 600. No crossover was evident in the changes in the fApEn curve with r that reflected the good relative consistency of fApEn in short physiological signals. Figures 4C,D depict the average change of EEG fApEn with increase of N and r of 11 stroke subjects at six electrodes (C3, C4, FC3, FC4, CP3, and CP4). They have the same pattern as S3 in Figure 4A. Combining the results of a previous study (Ao et al., 2015) with those testing results of the present study, N was set at 1000, and r was fixed at 0.2. Figure 4B depicts average multiscale fApEn of EEG of 11 stroke subjects at six electrodes. Based on the sampling rate of the EEG data (256 Hz), fApEn was calculated at time scale from 1 to 20. fApEn increase after training at fine time scales at all of the six electrodes. However, the changes of fApEn at course time scales are not consistent at six electrodes. So, we used fApEn at fine time scale (time scales = 1) to analysis EEG data in this study.

**Figure 5:**
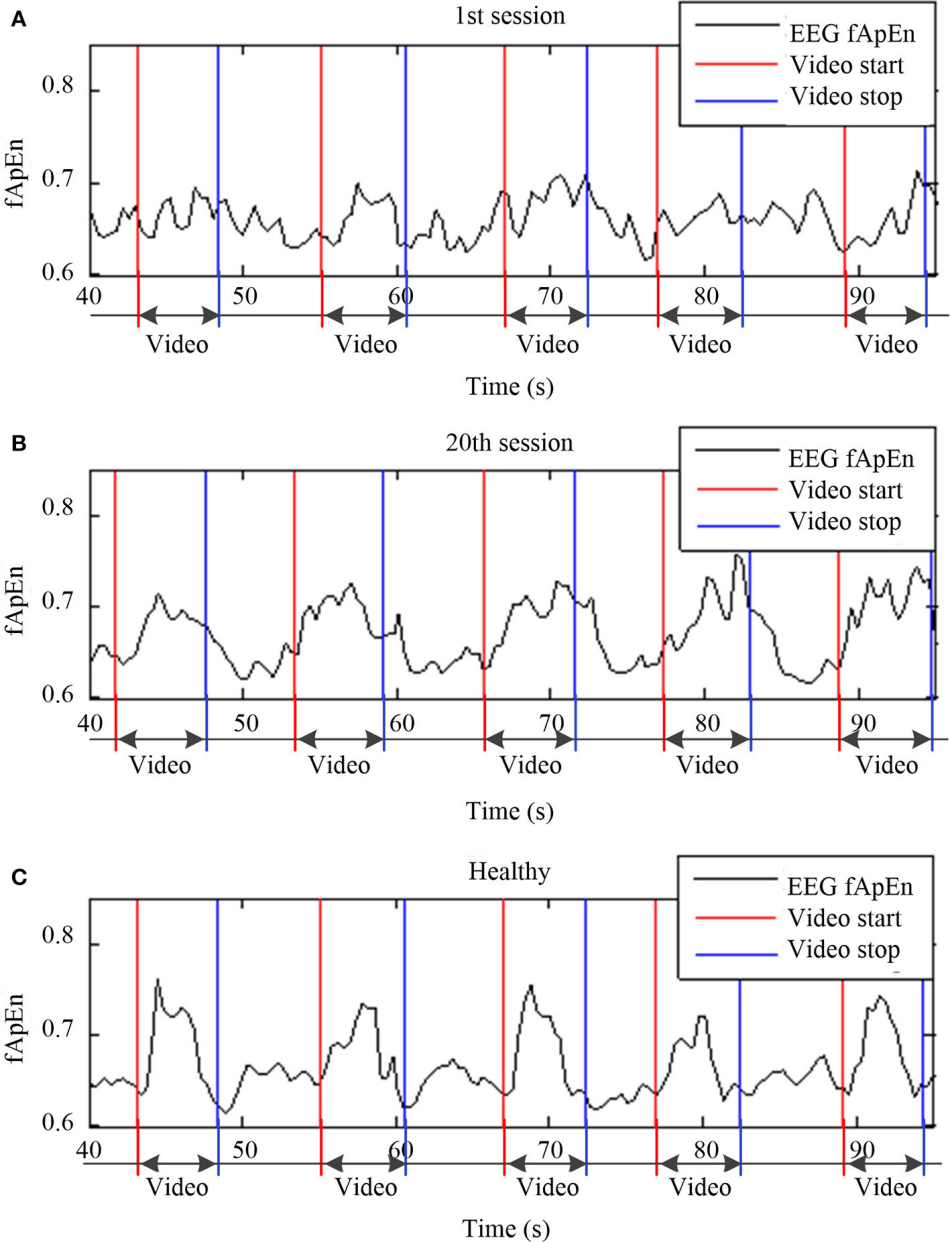
Sliding window of EEG fApEn during the **(A)** first training session, **(B)** and 20th training session from a stroke participant (S3) and a healthy participant **(C)**.

To obtain a continuous fApEn curve, a sliding window was applied during EEG data processing. The moving step was 0.1 s (i.e., 26 points). Figure 5 shows the comparison of sliding-window EEG fApEn during the 1st (Figure 5A) and 20th training sessions (Figure 5B) from a stroke participant (S3) and a healthy subject (Figure 5C). The period of the video clip was represented between the red line and the blue line. The fApEn curve showed no clear trend in changes, and the values recorded during the video clip showed no remarkable characteristics during the 1st session of training. After training, the fApEn curve showed clear peaks during the period of the video clip, and the fApEn value was relatively low during segments without a video clip, consistent with the shape of the EEG fApEn curve from the healthy participant.

**Figure 6:**
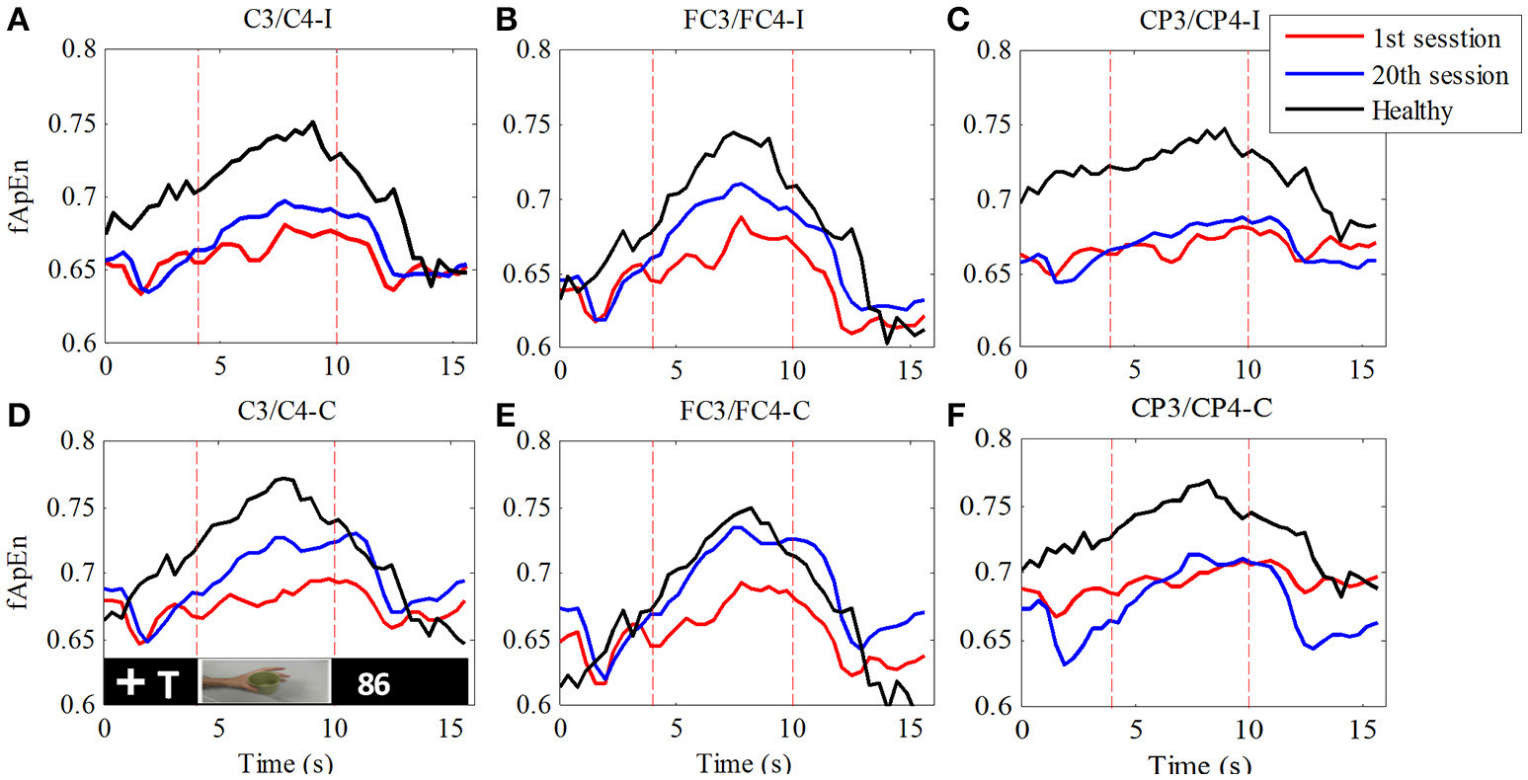
Comparison of fApEn during observation of biological movement at six electrodes (**A**, C3/C4-ipsilesional; **B**, FC3/FC4-ipsilesional; **C**, CP3/CP4-ipsilesional; **D**, C3/C4-contralesional; **E**, FC3/FC4-contralesional; **F**, CP3/CP4-contralesional) among healthy participants and stroke participants, pre- and post-training.

In order to verify the change in the fApEn curve before and after training as illustrated in Figure 5, we separated all trials in each experiment by time-marks. Thus, the average of 100 trials in each training session was calculated. Figure 6 compares the average fApEn between the 1st and 20th training sessions among 11 stroke participants. In addition, Figure 6 presents the average EEG fApEn generated from nine healthy participants. The EEG fApEn before the observation of the action was relatively low. When the video clip was started, the fApEn increased significantly, indicating an increase in the complexity of the EEG over the primary motor cortex. When biological observation tasks were complete, the EEG fApEn was reduced to the baseline level (Figure 6). Furthermore, after training, the fApEn during the video clip was higher than that before training. The fApEn during the video clip was also closer to that of the fApEn in healthy study participants, in comparison to the fApEn before training.

**Figure 7:**
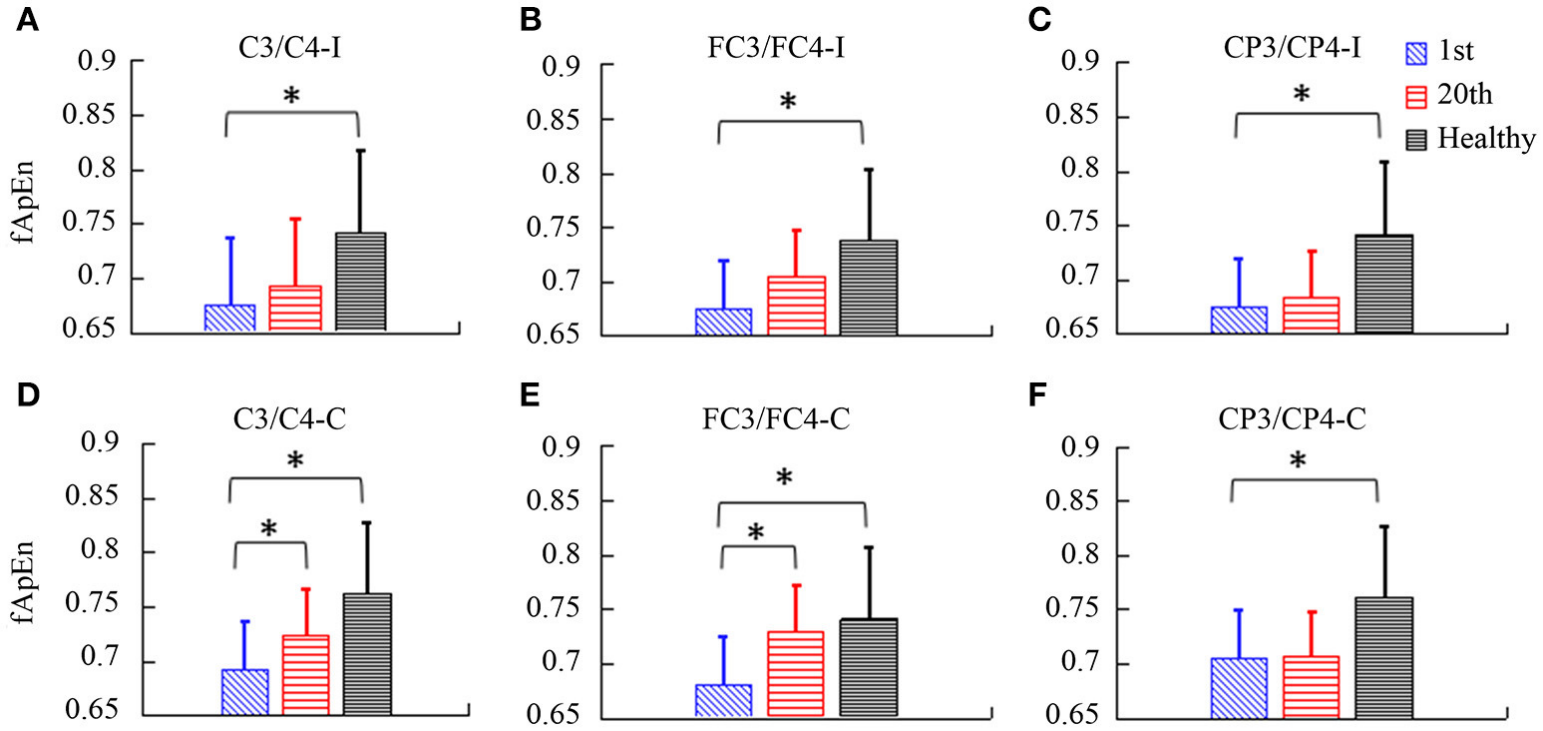
The fApEn values among healthy participants and stroke participants during observation of biological movements at six electrodes, pre- and post-training (**A**, C3/C4-ipsilesional; **B**, FC3/FC4-ipsilesional; **C**, CP3/CP4-ipsilesional; **D**, C3/C4-contralesional; **E**: FC3/FC4-contralesional; **F**, CP3/CP4-contralesional). ^*^p < 0.05

fApEn during the period of the video clip was averaged for statistical analysis. In Figure 7, there was a significant interaction between the time and electrode, *F*_(2.49, 24.85)_ = 3.432, *p* = 0.039. The fApEn of C3/C4-contralesional and FC3/FC4-contralesional (C3/C4-contralesional: *F* = 5.407, *p* = 0.042; FC3/FC4-contralesional: *F* = 7.107, *p* = 0.024) increased after training. However, there was no significant main effect of the time [*F*_(1, 10)_ = 4.29, *p* = 0.065], and the electrode [*F*_(1.88, 18.79)_ = 1.60, *p* = 0.229]. There was a significant difference in fApEn of the electrodes between healthy controls and stroke patients before the training, *F*_(1, 17)_ = 7.68, *p* = 0.013. The fApEn of all electrodes in healthy controls were larger than those in stroke patients. However, there was no significant main effect of the electrode [*F*_(2.30, 39.07)_ = 0.971, *p* = 0.398], and the electrode^*^group interaction [*F*_(2.30, 39.07)_ = 0.991, *p* = 0.390]. The covariate, age, was not significantly related to the fApEn, *F*_(1, 17)_ = 2.66, *p* = 0.121. There was no significant difference in fApEn of the electrodes between healthy controls and stroke patients after the training, *F*_(1, 17)_ = 3.95, *p* = 0.063. There was also no significant main effect of the electrode [*F*_(1.72, 29.18)_ = 0.16, *p* = 0.822], and the electrode^*^group interaction [*F*_(1.72, 29.18)_ = 0.29, *p* = 0.720]. The covariate, age, was not significantly related to the fApEn, *F*_(1, 17)_ = 1.60, *p* = 0.224.

### Correlation of fApEn with clinical scales in stroke patients

**Figure 8:**
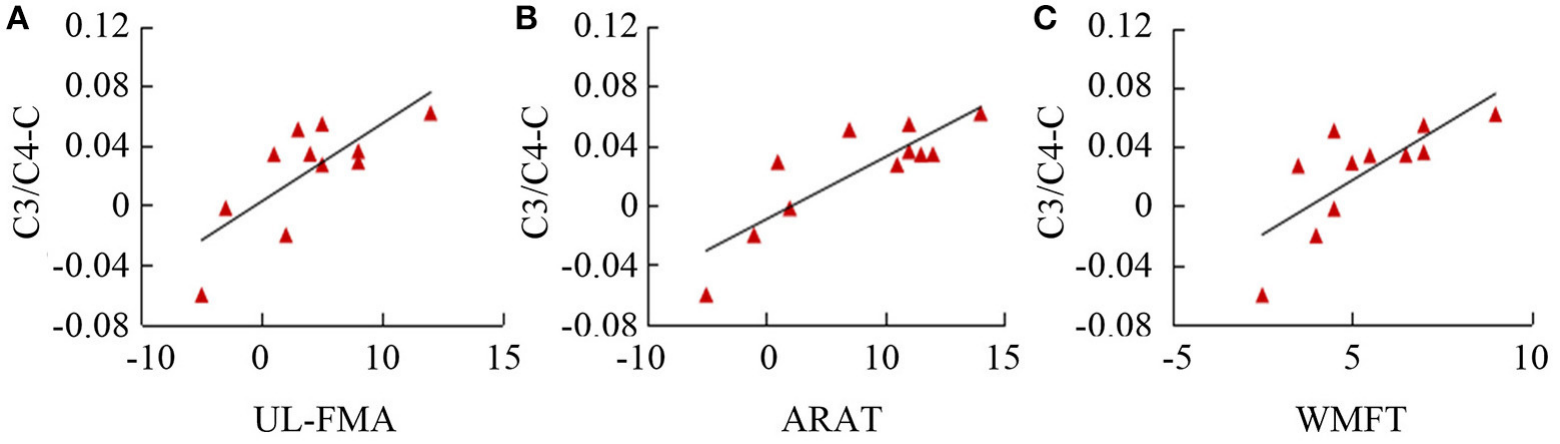
Correlation of fApEn with clinical scales (**A**, UL-FMA; **B**, ARAT; and **C**, WMFT) in stroke patients.

Table 3 summarizes the results of Spearman's correlation analyses performed between fApEn and the clinical scales (UL-FMA, ARAT, and WMFT) used in the stroke patients. For the EEG fApEn of the ipsilesional hemisphere, no significant correlation was noted between the changes observed with the clinical scales (post- minus pre-training) and those of the average fApEn in the electrodes (*p* > 0.05). For the EEG fApEn of the contralesional hemisphere, a significant correlation was observed between the change in fApEn in the C3/C4-contralesional hemisphere and the change reflected by the UL-FMA (*R* = 0.671, *p* = 0.024); ARAT (*R* = 0.774, *p* = 0.005); and WMFT (*R* = 0.849, *p* = 0.001), as shown in Figures 8A-C respectively. However, the fApEn in the FC3/FC4-contralesional and CP3/CP4-contralesional hemispheres showed no correlation with changes reflected by any of the clinical scales.

**Table 3:** Correlations between change in clinical scales (UL-FMA, ARAT, WMFT) and change in EEG fApEn at six electrodes (C3, C4, FC3, FC4, CP3, CP4).

	**UL-FMA**	**ARAT**	**WMFT**
C3/C4 I.	*R* = −0.023; *p* = 0.947	*R* = 0.155; *p* = 0.649	*R* = −0.050; *p* = 0.883
C3/C4 C.	*R* = 0.671; *p* = 0.024^*^	*R* = 0.774; *p* = 0.005^**^	*R* = 0.849; *p* = 0.001^**^
FC3/FC4 I.	*R* = −0.064; *p* = 0.852	*R* = 0.036; *p* = 0.915	*R* = −0.032; *p* = 0.926
FC3/FC4 C.	*R* = 0.511; *p* = 0.108	*R* = 0.574; *p* = 0.065	*R* = 0.516; *p* = 0.104
CP3/CP4 I.	*R* = 0.201; *p* = 0.554	*R* = 0.292; *p* = 0.384	*R* = −0.237; *p* = 0.482
CP3/CP4 C.	*R* = 0.498; *p* = 0.119	*R* = 0.346; *p* = 0.297	*R* = 0.315; *p* = 0.345

*I., ipsilesional hemisphere; C., contralesional hemisphere; UL-FMA, Upper Limb Fugl-Meyer Assessment; ARAT, Action Research Arm Test; WMFT, Wolf Motor Function Test*.

* p < 0.05;

** *p < 0.01*.

## Discussion

### The effect of BCI-motor observation training

To answer the question whether the motor function of the upper limb recovery after BCI-motor observation training in post-stroke hemiparetic patients, this study adopted motor observation in BCI rehabilitation, and then tested the effects of training. Both the WMFT and ARAT demonstrated significant improvement in upper limb motor function after training. These findings indicate that the novel training system improved motor function of the upper limb.

Several studies (Jeannerod and Frak, 1999; Jackson et al., 2001; Page, 2001; Sharma et al., 2006) suggest that motor imagery could be a useful training method for stroke rehabilitation; however, one study (Ietswaart et al., 2011) reported mental practice with motor imagery does not enhance motor recovery on the ARAT. Differences in understanding of motor imagery in different individuals might be the main reason for these disparities. We believe that motor observation could overcome the challenges associated with the interpretation of motor imagery, because it is a passive activity with smaller variations among individuals, in comparison to voluntary motor imagery. On the other hand, motor observation could activate a similar region in the cortex as motor imagery and execution. Maeda et al. (Maeda et al., 2002) demonstrated the involvement of the primary motor cortex during action observation, indicating similarity not only between action and imagery but also between action and observation. The study by Maeda et al. also shows that “passive” movement observation, as distinct from that with “the intention to imitate,” activates structures in the brain that are normally involved in the planning and execution of movements, including supplementary motor area (SMA), the premotor cortex, the superior temporal sulcus, the inferior frontal cortex (area 45), and the inferior parietal cortex (area 40). Hence, an active intention to imitate does not seem to be crucial for the observation of movement-related neural activity in motor areas. In the present study, whether or not a subject performed motor imagery during motor observation was not a crucial point, because passive motor observation could also activate similar brain cortical activity as motor imagery.

### The fApEn of EEG during observation of biological movements

A dynamic nonlinear index, EEG complexity, was used to describe EEG oscillation during motor observation. According to previous reports, the fApEn shows good consistency and robustness to noise in the evaluation of a complexity of bio-signals, and a very short data length (0.5 s) is needed to distinguish different processes (Ao et al., 2015). In this study, the fApEn increased during observation of biological movement and was reduced to baseline levels during observation of non-biological movement. This could be attributed to activation of the motor cortex during motor observation. During the mental activity, different neuronal networks at the cortical level start to oscillate at different frequencies, and this can reduce the predictability of the EEG signal. However, during the resting state, a relatively large number of neuronal networks fire synchronously, thereby increasing the predictability of the signal. Ming et al. (2009) found that MI and rest can be discriminated with the use of multiscale entropy in the EEG. Furthermore, they reported that the entropy of MI is significantly higher than that at rest, which is consistent with the present results. A key difference is that the present study used fApEn, whereas Ming's study used multiscale entropy. Both of these algorithms can be used to evaluate EEG complexity.

### The fApEn of EEG from healthy adults and stroke patients, pre- and post-treatment

To our knowledge, this is the first report of an investigation into the dynamic temporal complexity of EEG, using fApEn in post-stroke patients and healthy adults during BCI-motor observation training. The results show that fApEn values of post-stroke patients were significantly lower than those of healthy adults in the fronto-central, central regions pre-training. This suggests that the motor dysfunction of post-stroke patients is characterized by a loss of complexity of brain activity. However, the associated pathophysiological implications remain unclear. These findings might be attributed to neuronal death, loss of synaptic connections, and the general effects of neurotransmitter deficiency. These results also highlight the possibility that fApEn could be used as a novel evaluation to facilitate the diagnosis of brain disorders such as stroke by qualifying brain activity.

The low EEG complexity in stroke patients was increased to the level of healthy subjects in the fronto-central region after training. In contrast to the reduction in fApEn observed after stroke possibly due to neuronal death, the increase in fApEn after treatment could not have been due to an increase in the number of neurons, as lost neurons cannot be regenerated during rehabilitation training. However, following the neuronal death in stroke, spared neural structures in adjacent tissue, and remote structures in the ipsilesional and contralesional hemispheres, undergo significant functional changes (Nudo and Hillis, 2010). For example, changes in neural pathways in remote areas of the brain occur, and new neuronal connections develop during rehabilitation (Pekna et al., 2012) that could have led to the increased complexity of brain signals.

The extent of the restoration of motor function is highly dependent on remapping in the uninjured motor-related areas apart from the site of the lesion in the brain (Murphy and Corbett, 2009). The present results indicate that the EEG complexity increases much more significantly in the contralesional hemisphere than in the ipsilesional hemisphere, particularly in the fronto-central region. This indicates that the contralesional hemisphere can become more active after the training. In animal studies, a greater number of distant motor-related sites, such as contralesional hemisphere, become involved if a stroke has induced a relatively large lesion (Brown et al., 2009). The region of the brain injury in all subjects of the present study was relatively large, so there might have been insufficient cortical tissue left in the ipsilesional hemisphere to re-establish a new motor center. Also, the majority of our participants had the left hand affected, suggesting a lesion in the non-dominant right hemisphere. The increase of EEG complexity in contralesional hemisphere may due to the dominant left hemisphere (contralesional for these patients) exerts a stronger influence on the right hemisphere's motor activity than the other way around (Derakhshan, 2005).

### The correlation between fApEn and clinical scales

The trend of an increase in the group means of clinical scales (FMA-UL, ARAT, and WMFT) across the training sessions suggest an improvement of motor function in the paretic limbs. The fApEn value increased as rehabilitation training progressed, and this might have been caused by changes in neuronal pathways. According to a previous study, neurophysiologic and neuroanatomic changes that take place in undamaged tissue could be explained by the functional plasticity of the adult cerebral cortex, even as training sessions proceed (Nudo et al., 2001). Moreover, the significant correlation between clinical scales and the fApEn of the EEG implied that modulation of the neural pathway is one of the factors responsible for the improvement in motor function. It is interesting to note that, among six electrodes, only the fApEn in the central region (C3/C4) of the contralesional hemisphere showed significant correlation with clinical scales. Coincidentally, a significant increase in the fApEn of this region was noted after the training.

### Prospects, limitations, and future plans

This study provides new information about electrophysiological aspects based on the fApEn of EEG signals. This information is both useful for, and complementary to, the clinical evaluation of motor status. Moreover, the method outlined is very simple, as motor observation can be easily performed and the subject experiences no associated discomfort. The method and findings of the present study have clinical potential for the evaluation of improvement in the motor system and for evaluation of the effects of therapeutic devices or rehabilitative therapies. However, this paper represents a pilot study on BCI-motor observation training. Future studies that evaluate a greater number of patients and healthy subjects will be necessary to verify the conclusions of the present study.

## Author contributions

RS designed, performed experiments, analyzed data, and wrote the paper; RT designed, performed experiments and wrote the paper; WW performed experiments; JW designed and performed experiments; all authors reviewed the manuscript.

### Conflict of interest statement

The authors declare that the research was conducted in the absence of any commercial or financial relationships that could be construed as a potential conflict of interest.

## Abbreviations

fApEn: Fuzzy Approximate Entropy
BCI: Brain Computer Interface
EEG: Electroencephalography
FFT: Fast Fourier Transform
MI: Motor Imagery
MNS: Mirror neuron system
SE: Standard error
WMFT: Wolf Motor Function Test
ARAT: Action Research Arm Test
FMA-UL: Fugl Meyer Assessment-Upper Limb.
